# The efficacy and safety of addition of pegylated interferon to long-term nucleos(t)ide analogue therapy on functional cure of chronic hepatitis B patient: a systematic review and meta-analysis

**DOI:** 10.3389/fphar.2024.1474342

**Published:** 2024-10-31

**Authors:** Xu Zhang, Xianzhao Yang, Lingjie Tan, Yujia Tian, Zhiren Zhao, Shuying Ru

**Affiliations:** ^1^ Dongzhimen Hospital, Beijing University of Chinese Medicine, Beijing, China; ^2^ Tongzhou District of Dongzhimen Hospital, Beijing University of Chinese Medicine, Beijing, China

**Keywords:** pegylated interferon, nucleos(t)ide analogues, chronic hepatitis B, functional cure, meta-analysis

## Abstract

**Objective:**

This meta-analysis aims to assess the efficacy and safety of adding pegylated interferon (Peg-IFN) to long-term nucleos(t)ide analogs (NAs) treatment for achieving functional cure in patients with chronic hepatitis B (CHB).

**Methods:**

This meta-analysis was registered in PROSPERO (CRD42024519116). We searched PubMed, Embase, Cochrane Library and Web of Science for randomized controlled trials that compared adding Peg-IFN to long-term NAs with NAs alone for the treatment of CHB. Relative risks (RR) and 95% confidence interval (CI) were pooled using a random-effects model.

**Results:**

Seven trials with 692 participants were included. Compared to NAs monotherapy, sequential combination therapy significantly increased the HBsAg seroclearance rate (RR 4.37, 95%CI: 1.92–9.55; I^2^ = 0%) and HBsAg seroconversion rate (RR 3.98, 95%CI: 1.50–10.54; I^2^ = 0%), and the results reached statistical significance. Compared to NAs monotherapy, sequential combination therapy showed a significant increase in HBeAg seroclearance rate (RR 2.04; 95%CI: 0.47–8.82; I^2^ = 73%) and HBeAg seroconversion rate (RR 2.10; 95%CI: 0.41–10.71; I^2^ = 67%), but did not reach statistical significance. Sequential combination therapy was more likely to experience adverse events. Although most reactions are mild and reversible, vigilant monitoring for treatment-related adverse events is essential, with prompt intervention when needed.

**Conclusion:**

For CHB patients on long-term NAs treatment, sequential combination therapy boosts HBsAg seroclearance and HBsAg seroconversion rates compared to monotherapy. However, it may increase adverse events. Additional studies are needed to thoroughly evaluate its clinical effectiveness, given the current limited research available.

**Systematic Review Registration:**

PROSPERO, identifier CRD42024519116.

## 1 Introduction

Chronic hepatitis B (CHB) is a persistent liver disease caused by hepatitis B virus (HBV) infection. Hepatitis B is a major global health problem, and the elimination of CHB is a significant challenge (World Health Organization, 2024a). Globally, approximately 296 million people are affected by chronic HBV infection (Hsu et al., 2023), and the World Health Organization (WHO) estimates 1.2 million new HBV infections annually. HBV is also the leading cause of cirrhosis and liver cancer worldwide (World Health Organization, 2024b). In 2019, HBV-related cirrhosis led to about 331,000 deaths, while deaths from HBV-related liver cancer in the same year numbered around 192,000, up from 156,000 in 2010 (Hsu et al., 2023).

Active treatment of hepatitis B virus can delay the progression of related diseases. Studies indicate that HBsAg seroclearance correlates with reduced risks of hepatic decompensation, cirrhosis, hepatocellular carcinoma (HCC), and improved prognosis (Kim et al., 2014; Yip et al., 2023). Achieving HBsAg seroclearance, known as a “functional cure,” is seen as a clinical treatment goal of CHB (Sarin et al., 2016; European Association for the Study of the Liver, 2017; Terrault et al., 2018). Currently, nucleos(t)ide analogues and (pegylated) interferons are the primary treatments for CHB (European Association for the Study of the Liver, 2017). Nucleoside analogues (NAs) effectively suppress viral replication long-term but achieving sustained immune control is challenging, often leading to high rates of virological relapse post-treatment (Hall et al., 2021). Interferon has antiviral and immune-modulating effects, but its use is limited by adverse reactions and tolerability issues, and the overall efficacy when used alone is relatively low (Terrault et al., 2018). With the continuous exploration of various mechanisms and drug targets, some progress has been made in the development of new drugs for hepatitis B (e.g., therapeutic vaccines, small-interfering RNAs, Capsid inhibitors, *etc.*), but they are seldom effective and durable in reducing HBsAg (Wong et al., 2022; Dusheiko et al., 2023). Consequently, before the discovery of more effective antiviral drugs, the focus of current clinical research is on combining these drugs to achieve the best clinical efficacy. Some studies (Hu et al., 2022; Farag et al., 2024) have shown that adding pegylated interferon (Peg-IFN) to long-term nucleoside analogue (NA) treatment in CHB patients can help achieve functional cure. However, this treatment strategy remains controversial, with EASL guidelines currently not recommending this combination for patients under long-term NA suppression (European Association for the Study of the Liver, 2017). This meta-analysis aimed to assess the efficacy and safety of adding pegylated interferon to long-term nucleos(t)ide analogue treatment for achieving a functional cure in CHB patients, providing insights for clinical decision-making.

## 2 Methods

### 2.1 Protocol and registration

The meta-analysis followed the guidelines outlined in the Cochrane Handbook for Systematic Reviews of Interventions and adhered to the PRISMA (Preferred Reporting Items for Systematic Reviews and Meta-Analyses) statement (Page et al., 2021). It was prospectively registered in the PROSPERO database (CRD42024519116).

### 2.2 Data sources

We comprehensively and systematically searched PubMed, Embase, Cochrane Library and Web of Science from inception to 13 October 2024. Our search was restricted by the English language. The search terms mainly included: chronic hepatitis b, hepatitis b, nucleos(t)ide analogues, nucleotide analog*, nucleoside analog*, peginterferon, pegylated interferon. Detailed search strategies are provided in Supplementary Table S1. Furthermore, references of relevant review articles and included studies were hand-searched to identify additional eligible studies.

### 2.3 Inclusion criteria

The meta-analysis included studies that met the following PICOS criteria:

Population: adult patients with chronic hepatitis B treated with nucleos (t) ide analogues for at least 12 months,
Intervention: add pegylated interferon to long-term nucleos(t)ide analogues therapy (sequential combination therapy),
Comparison: nucleos(t)ide analogs, Main outcomes: rates of HBsAg seroclearance or seroconversion at the end of treatment follow-up,
Study design: randomized controlled trials (RCTs).

### 2.4 Exclusion criteria

Patients coinfected with Human Immunodeficiency Virus (HIV), Hepatitis C Virus (HCV), or Hepatitis D Virus (HDV); patients with decompensated liver disease, hepatocellular carcinoma, or other serious liver and kidney diseases; patients who have undergone liver transplantation; patients with autoimmune diseases or metabolic liver disease; women who were pregnant or lactating; patients receiving corticosteroids, immunosuppressants, or Chinese herbal medicine concurrently.

Republished studies, unavailable full texts, unpublished or original data, or publications not in English.

### 2.5 Study selection

Initially, all retrieved literature was imported into Endnote software. Duplicate citations were removed using Endnote’s deduplication function and manual checks. Titles and abstracts were then reviewed to exclude irrelevant studies. Finally, full-text articles meeting the criteria were included in the analysis.

### 2.6 Data extraction

We used a standardized Excel sheet for data extraction. Two authors independently extracted data from each trial, resolving discrepancies through consensus. Extracted information included: first author, year of publication, study country, sample size, treatment regimen, follow-up duration, outcomes and adverse events. Primary efficacy outcomes were HBsAg seroclearance and seroconversion rates, while secondary outcomes included HBeAg seroclearance and seroconversion rates.

### 2.7 Quality assessment

We assessed the risk of bias using the Cochrane Risk of Bias tool for RCTs (Higgins et al., 2011). This tool evaluates randomization methods, allocation concealment, performance and detection biases, attrition and reporting biases, as well as other potential biases.

### 2.8 Statistical analysis

We calculated risk ratios (RRs) with 95% confidence intervals (CIs) for dichotomous outcomes. Meta-analyses employed a random-effects model to accommodate clinical heterogeneity. Statistical heterogeneity among trials was assessed using the Cochrane Q test (P< 0.1 indicating significance) and quantified with the I^2^ statistic (I^2^>50% indicating significant heterogeneity). A two-sided P< 0.05 was considered statistically significant. RevMan 5.3 from the Cochrane Collaboration was used for all statistical analyses. Publication bias was not assessed due to fewer than 10 included studies per outcome.

## 3 Results

### 3.1 Study selection

**FIGURE 1:**
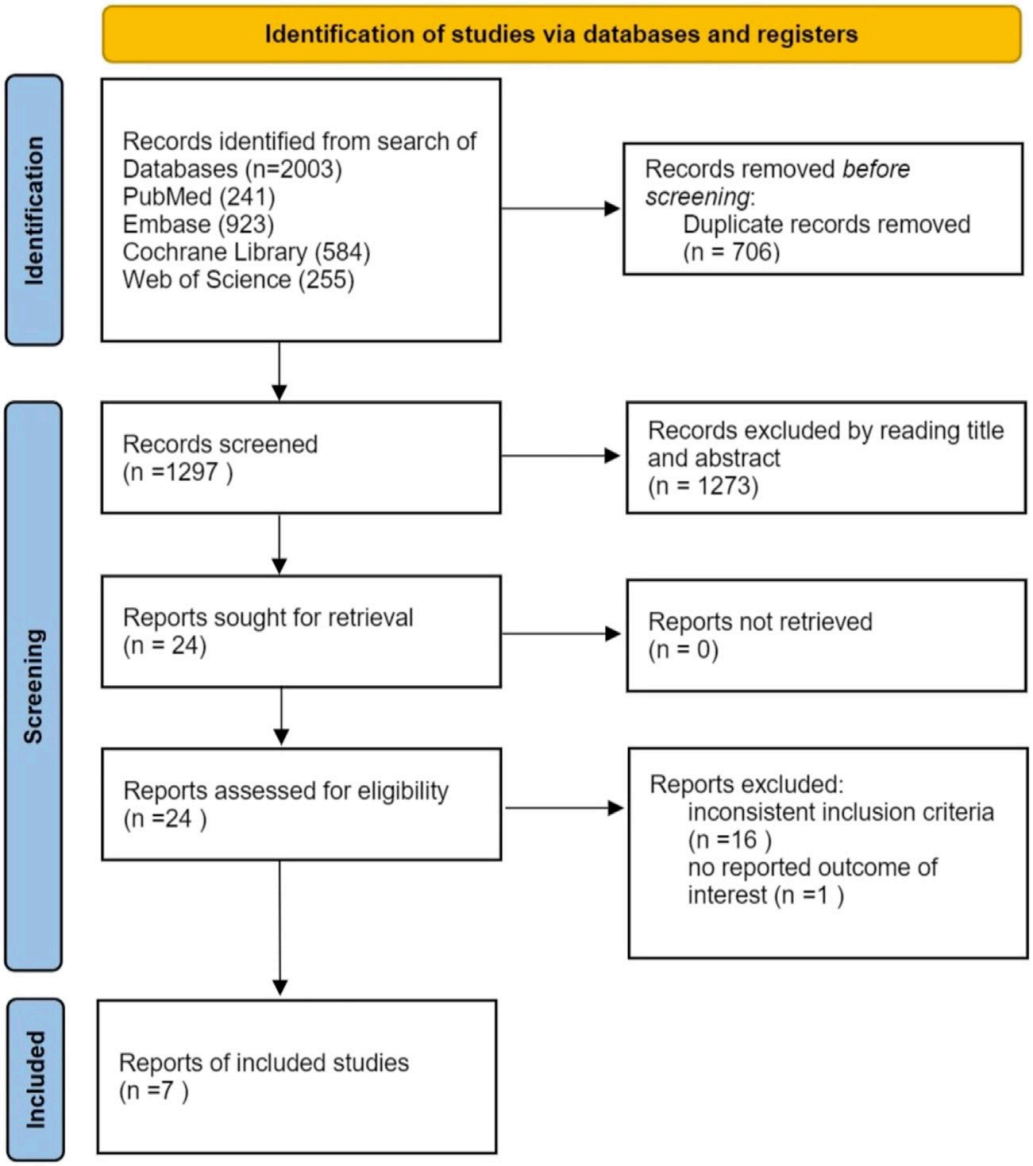
Study selection flow diagram.

A total of 2003 articles were initially retrieved from electronic databases. After removing duplicates, 1,297 articles underwent screening at the title and abstract level. Among these, 24 articles were assessed in full text. Subsequently, 17 articles were excluded: 16 did not meet the inclusion criteria, and 1 lacked relevant data. Finally, 7 trials were included (Farag et al., 2024; Bourlière et al., 2017; Cannizzo et al., 2018; Hu et al., 2022; Li et al., 2021; Lim et al., 2022; Vecchi et al., 2024). The study selection process is illustrated in Figure 1.

### 3.2 Study characteristics

A total of 692 participants were included across the studies (Bourlière et al., 2017; Cannizzo et al., 2018; Li et al., 2021; Hu et al., 2022; Lim et al., 2022; Farag et al., 2024; Vecchi et al., 2024), with 363 randomized to NAs plus PEG-IFN and 329 randomized to NAs monotherapy. Intention-to-treat analyses were performed in all but two trials (Li et al., 2021; Vecchi et al., 2024). These studies were published between 2017 and 2024. Detailed study characteristics are provided in Table 1.

**TABLE 1:** Basic characteristics of the included studies.

Study id	Country	Sample size (add on/control)	Mean age,years (SD)		HBeAg status	Duration of NA treatment, months	Interventions		Duration of follow-up	Outcome
			Add on	Control			Add on	Control		
Bourlière et al. (2017)	France	183 (90/93)	48.4 (12.1)	46.9 (11.3)	Negative	≥12	NA + Peg-IFNα-2a 48w	NA 48w	144w	①②
Cannizzo et al. (2018)	Italy	30 (10/20)	48 (6.3)	56 (11.7)	Negative	≥36	TDF + Peg-IFNα 48w	TDF 48w	12w	①②
Li et al. (2021)	China	89 (27/62)	32.93 (5.65)	34.27 (7.78)	Positive	—	TDF 48w→TDF + Peg-IFNα 48w	TDF 96w	0w	①③④
Lim et al. (2022)	Singapore	150 (99/51)	50.2 (12.0)	50.0 (12.2)	Negative and positive	>12	NA + Peg-IFNα-2b 48w	NA 48w	24w	①②③④
Hu et al. (2022)	China	101 (50/51)	38.1 (9.9)	38.9 (8.4)	Positive	≥24	ETV + Peg-IFNα-2b 48w	ETV 48w	24w	①②③④
Farag et al. (2024)	Netherlands and Canada	86 (58/28)	49 (10)	46 (11)	Negative	>12	NA + Peg-IFNα-2a 48w	NA 48w	24w	①
Vecchi et al. (2024)	Italy	53 (29/24)	51.6 (32.0)	57.0 (21.3)	Negative	>24	NA + Peg-IFNα 48w	NA 48w	48w	①②

NA, nucleos(t)ide analogue; ETV, entecavir; TDF, tenofovir disoproxil fumarate; PEG-IFN-α, pegylated interferon alpha; ① HBsAg seroclearance rate; ② HBsAg seroconversion rate; ③ HBeAg seroclearance rate; ④ HBeAg seroconversion rate.

### 3.3 Quality assessment

**FIGURE 2:**
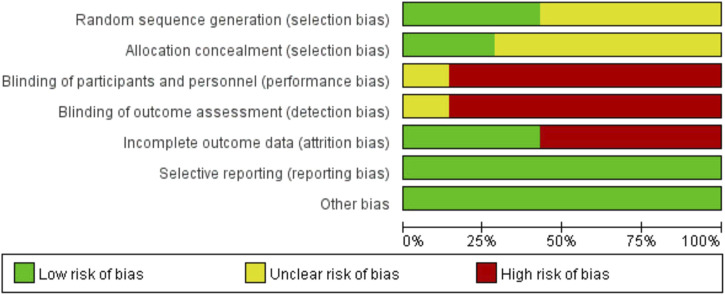
Risk of bias assessment.

**FIGURE 3:**
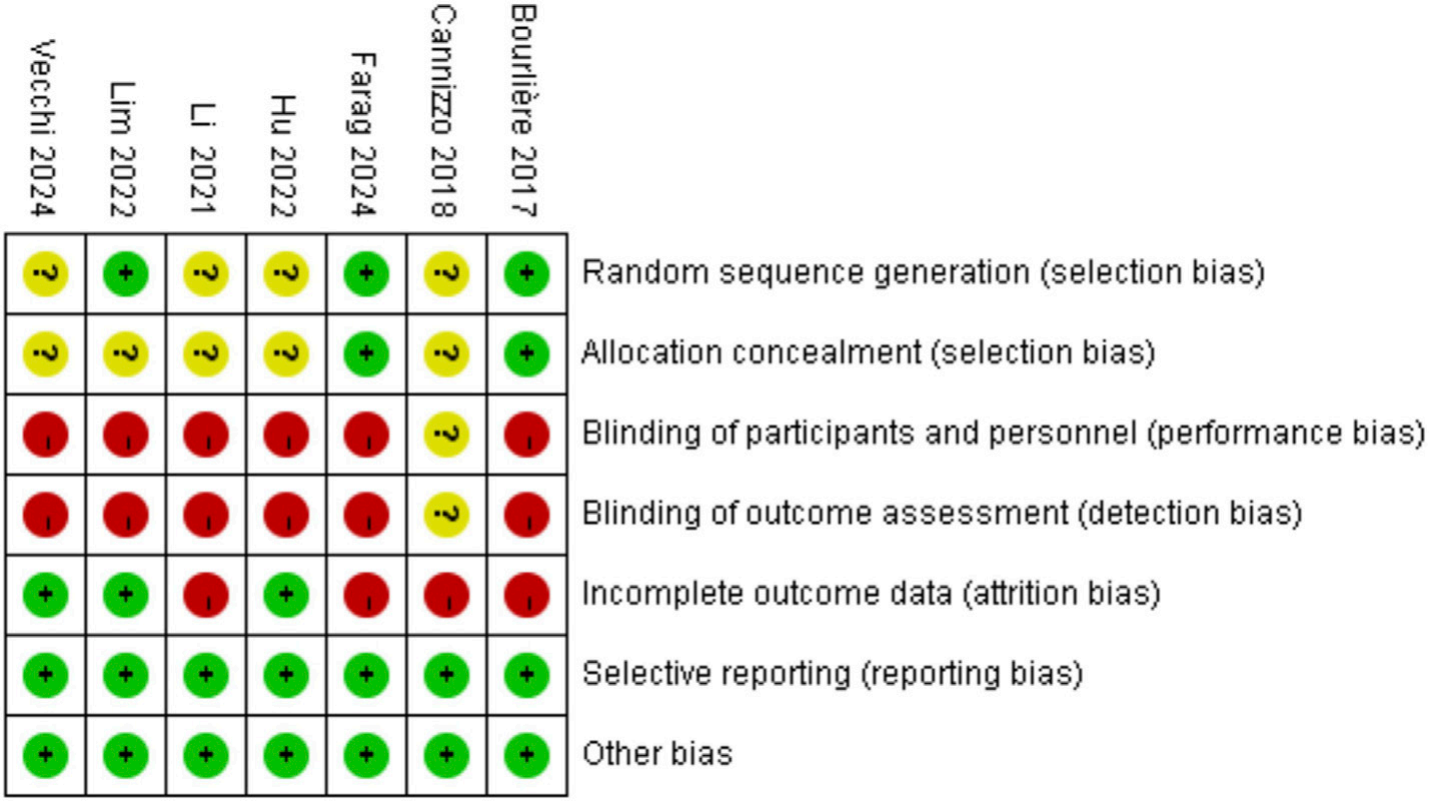
Risk of bias assessments on each study.

The quality of the included RCTs was assessed using the Rob tool, and the risk of bias in these studies is depicted in Figure 2. According to the Cochrane risk of bias assessment, all 7 RCTs (Bourlière et al., 2017; Cannizzo et al., 2018; Li et al., 2021; Hu et al., 2022; Lim et al., 2022; Farag et al., 2024; Vecchi et al., 2024) were classified as high risk. Detailed assessments for each trial across the 5 domains are presented in Figure 3.

### 3.4 HBsAg seroclearance rate

**FIGURE 4:**
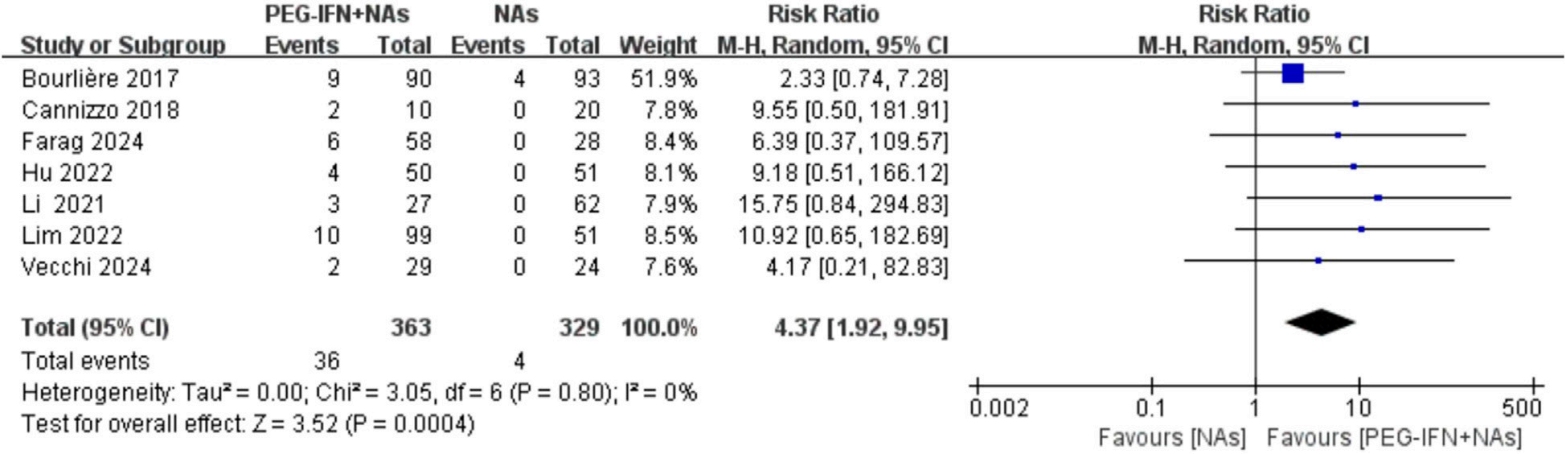
Forest plot for HBsAg seroclearance rate.

All included trials reported on HBsAg seroclearance rates (Bourlière et al., 2017; Cannizzo et al., 2018; Li et al., 2021; Hu et al., 2022; Lim et al., 2022; Farag et al., 2024; Vecchi et al., 2024). Compared to nucleos(t)ide analog monotherapy, sequential combination therapy significantly increased the HBsAg seroclearance rate (RR 4.37, 95% CI: 1.92–9.95; *p* = 0.0004; I^2^ = 0%; Figure 4). Sensitivity analysis, conducted by excluding each individual study, demonstrated no statistically significant changes in the results, confirming the robustness of the findings.

### 3.5 HBsAg seroconversion rate

**FIGURE 5:**
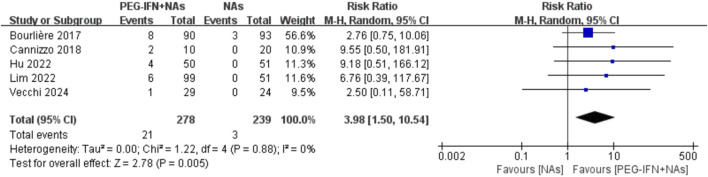
Forest plot for HBsAg seroconversion rate.

Five trials included in the analysis reported on the HBsAg seroconversion rate (Bourlière et al., 2017; Cannizzo et al., 2018; Hu et al., 2022; Lim et al., 2023; Vecchi et al., 2024). Compared to nucleos(t)ide analog monotherapy, sequential combination therapy significantly increased the HBsAg seroconversion rate (RR 3.98, 95% CI: 1.50–10.54; *p* = 0.005; I^2^ = 0%; Figure 5). Sensitivity analysis, where each individual study was sequentially excluded, showed no statistically significant changes in the results, indicating the robustness of the findings.

### 3.6 HBeAg seroclearance rate

**FIGURE 6:**
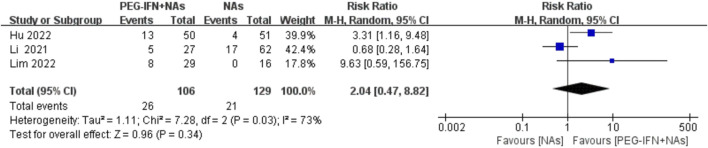
Forest plot for HBeAg seroclearance rate.

Three trials included in the analysis reported the rate of HBeAg seroclearance (Li et al., 2021; Hu et al., 2022; Lim et al., 2022). Compared to nucleos(t)ide analog monotherapy, sequential combination therapy showed a significant increase in HBeAg seroclearance, but the results did not reach statistical significance (RR 2.04; 95% CI: 0.47–8.82; *p* = 0.34; I^2^ = 73%; Figure 6). The meta-analysis results may be sensitive to variations in the number of studies, suggesting caution in interpretation due to potential lack of robustness.

### 3.7 HBeAg seroconversion rate

**FIGURE 7:**
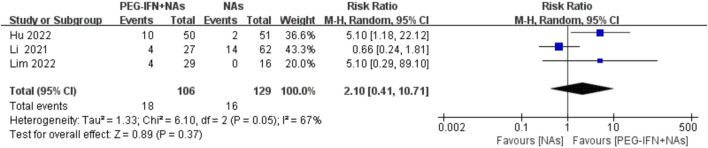
Forest plot for HBeAg seroconversion rate.

Three trials included in the analysis reported the rate of HBeAg seroconversion (Li et al., 2021; Hu et al., 2022; Lim et al., 2022). Compared to nucleos(t)ide analog monotherapy, sequential combination therapy showed an increase in HBeAg seroconversion rate, but the results did not reach statistical significance (RR 2.10; 95% CI: 0.41–10.71; *p* = 0.37; I^2^ = 67%; Figure 7). The meta-analysis results may be sensitive to variations in the number of studies, indicating potential lack of robustness, and thus should be interpreted with caution.

### 3.8 Adverse reactions

Three studies (Hu et al., 2022; Lim et al., 2022; Farag et al., 2024) indicate that patients receiving additional PEG-IFN treatment are more likely to experience adverse events compared to those on NA monotherapy. Common adverse events include fatigue, headache, myalgia, flu-like syndrome, neutropenia, thrombocytopenia, and elevated ALT levels. However, most reactions were mild and reversible. Among these studies, severe interferon-related adverse events that did not result in death were reported in the PEG-IFN group but not in the NA monotherapy group, according to Cannizzo et al. (2018), Li et al. (2021) noted one adverse event in the sequential combination group, contrasting with no adverse events in the monotherapy group. Bourlière et al. (2017) highlighted significant declines in physical and mental health-related quality of life, fatigue impact scale, and self-reported symptoms during Peg-IFN therapy, although these measures returned to baseline values by week 96 compared to the monotherapy group.

## 4 DISCUSSION

### 4.1 Main findings

Our meta-analysis reviewed current literature comparing sequential combination therapy with NAs monotherapy for CHB treatment. It found that sequential combination therapy increased HBsAg seroclearance or seroconversion rates compared to NAs alone. However, patients on sequential combination therapy were more likely to experience adverse events like fatigue, headache, myalgia, flu-like syndrome, neutropenia, thrombocytopenia, and abnormal ALT levels, although most were mild and reversible. A few patients in the sequential therapy group also experienced severe adverse events related to Peg-IFN use, whereas none occurred in the nucleos(t)ide analog monotherapy group, and there were no fatalities.

### 4.2 Action mechanism

Antiviral therapy is crucial for treating CHB. Current major options include Peg-IFN and NAs (Sarin et al., 2016; European Association for the Study of the Liver, 2017; Terrault et al., 2018; World Health Organization, 2024b). NAs inhibit HBV DNA polymerase to suppress virus replication. However, achieving sustained virologic response requires long-term treatment, posing challenges such as drug resistance, and there is a high rate of virological relapse upon cessation (Ning et al., 2019; Hall et al., 2021). Interferon has antiviral and immune-modulating effects, but is limited by adverse reactions, tolerability issues, and lower efficacy when used alone (Terrault et al., 2018). Neither therapy directly targets cccDNA, complicating efforts to eliminate it completely (Terrault et al., 2018; Ning et al., 2019).

High HBsAg, HBeAg, and HBV DNA levels can induce immune tolerance, reducing response to Peg-IFN therapy (Op Den Brouw et al., 2009; Lebossé et al., 2017; Zheng et al., 2021; Zheng et al., 2022). Long-term NAs antiviral therapy effectively suppresses HBV DNA replication and lowers HBsAg levels, aiding immune recovery and enhancing response to immunomodulators like Peg-IFN (Boni et al., 2012; Lim et al., 2023). Previous studies have found that Peg-IFN can cause immune function activation in CHB patients, promoting HBsAg clearance (Zeng et al., 2020). Combining these treatments may synergistically enhance antiviral effects, potentially leading to more HBsAg clearance and supporting functional cure. Sequential combination therapy is thus hypothesized to optimize antiviral effects and improve treatment outcomes for CHB.

### 4.3 Comparison with existing review

Five previous reviews (Li et al., 2011; Kim et al., 2016; Zhang et al., 2016; Qiu et al., 2018; Slaets et al., 2020) have examined combination therapy with Peg-IFN and NAs for CHB. However, three of these studies (Li et al., 2011; Kim et al., 2016; Zhang et al., 2016) did not specify whether patients had received NAs prior to combination therapy, and two studies (Li et al., 2011; Zhang et al., 2016) found no significant difference in HBsAg seroclearance or seroconversion between combination therapy and Peg-IFN monotherapy. One study (Slaets et al., 2020) suggested that patients achieving HBV DNA suppression (from 2000 IU/mL to undetectable levels) with NAs may experience increased rates of HBsAg seroclearance upon adding or switching to Peg-IFN. Another study (Qiu et al., 2018) indicated higher rates of HBsAg seroclearance and seroconversion with sequential combination therapy, where NAs were initiated for at least 48 weeks before adding Peg-IFN, compared to simultaneous initiation of NAs and Peg-IFN.

Our meta-analysis focused on CHB patients treated with NAs for more than 12 months. We found that adding Peg-IFN significantly boosts HBsAg seroclearance or seroconversion rates, highlighting its role in achieving a functional cure for CHB patients. Importantly, our analysis also evaluated safety, noting a higher incidence of adverse events with the addition of Peg-IFN, including severe cases. Moreover, our meta-analysis incorporated the latest trials, making it the most current and comprehensive review to date, which reinforces previous findings. We utilized a random-effects model to accommodate clinical heterogeneity when summarizing the data.

### 4.4 Implication for clinical practice

HBsAg seroclearance is crucial for achieving long-term efficacy in CHB treatment, often viewed as the ideal therapeutic goal (Sarin et al., 2016; European Association for the Study of the Liver, 2017; Terrault et al., 2018; World Health Organization, 2024b). This study shows that adding Peg-IFN to long-term NAs treatment significantly improves HBsAg seroclearance or seroconversion rates, facilitating progress towards a functional cure. Promoting this approach in clinical practice is warranted. Sequential combination therapy was more likely to experience adverse events. Although most reactions are mild and reversible, vigilant monitoring for treatment-related adverse events is essential, with prompt intervention when needed.

### 4.5 Limitations

Our study has several limitations. Firstly, The number of literature included in the HBeAg clearance and seroconversion rate outcome metrics was too small for example, Li et al. (2021) accounted for a large weight, and the heterogeneity test I^2^ = 0 after removal,so the results should be interpreted with more caution. Secondly, it is challenging to rule out publication bias due to the small number of trials included (7 in total). Thirdly, variations in populations and treatment regimens may introduce clinical and methodological heterogeneity. Lastly, the analysis can only be based on the existing data, and whether HBsAg or HBeAg will reappear or loss in the future remains to be further discussed. Therefore, further validation of our conclusions is essential through larger sample sizes, longer follow-up periods and high-quality RCTs.

## 5 Conclusion

For CHB patients on long-term NAs treatment, sequential combination therapy boosts HBsAg seroclearance and serological conversion rates compared to monotherapy. However, it may increase adverse events. Additional studies are needed to thoroughly evaluate its clinical effectiveness, given the current limited research available.

## Data Availability

The original contributions presented in the study are included in the article/Supplementary Material, further inquiries can be directed to the corresponding authors.
