# Supplementary material for: The efficacy and safety of addition of pegylated interferon to long-term nucleos(t)ide analogue therapy on functional cure of chronic hepatitis B patient: a systematic review and meta-analysis
Source: Front Pharmacol. 2024 Oct 31;15:1474342. doi: 10.3389/fphar.2024.1474342 (PMC11560418; doi:10.3389/fphar.2024.1474342)
Supplement: Supplementary file 1 [file Table1.DOCX]

**The retrieval strategies and retrieval results of each database are shown in Tables 1-4**.

Table 1: PubMed 2024.10.13

| No. | Content | Result |
| --- | --- | --- |
| #1 | Search: ((("Hepatitis B, Chronic"[Mesh]) OR "Hepatitis B"[Mesh]) OR "Hepatitis B virus"[Mesh]) OR ((((((Chronic Hepatitis B Virus Infection[Title/Abstract]) OR (Chronic Hepatitis B[Title/Abstract])) OR (Hepatitis B Virus Infection, Chronic[Title/Abstract])) OR (Hepatitis B Virus Infection[Title/Abstract])) OR (CHB[Title/Abstract])) OR (HBV[Title/Abstract])) Sort by: Most Recent | 96,339 |
| #2 | Search: ("Nucleosides"[Mesh]) OR (((((((((((((((Nucleoside[Title/Abstract]) OR (Nucleotide analog*[Title/Abstract])) OR (Nucleoside analog*[Title/Abstract])) OR (NAs[Title/Abstract])) OR (NUC[Title/Abstract])) OR (Lamivudine[Title/Abstract])) OR (LAM[Title/Abstract])) OR (Adefovir[Title/Abstract])) OR (ADV[Title/Abstract])) OR (Entecavir[Title/Abstract])) OR (ETV[Title/Abstract])) OR (Telbivudine[Title/Abstract])) OR (TBV[Title/Abstract])) OR (Tenofovir[Title/Abstract])) OR (TDF[Title/Abstract])) Sort by: Most Recent | 304,313 |
| #3 | Search: ("Interferons"[Mesh]) OR (((((Peginterferon[Title/Abstract]) OR (pegylated interferon[Title/Abstract])) OR (PEG-IFN[Title/Abstract])) OR (pegasys[Title/Abstract])) OR (peg interferon[Title/Abstract])) Sort by: Most Recent | 150,966 |
| #4 | Search: (((randomized controlled trial[Title/Abstract]) OR (randomized[Title/Abstract])) OR (random*[Title/Abstract])) OR (placebo[Title/Abstract]) Sort by: Most Recent | 1,650,,866 |
| #4 | Search: (((((("Hepatitis B, Chronic"[Mesh]) OR "Hepatitis B"[Mesh]) OR "Hepatitis B virus"[Mesh]) OR ((((((Chronic Hepatitis B Virus Infection[Title/Abstract]) OR (Chronic Hepatitis B[Title/Abstract])) OR (Hepatitis B Virus Infection, Chronic[Title/Abstract])) OR (Hepatitis B Virus Infection[Title/Abstract])) OR (CHB[Title/Abstract])) OR (HBV[Title/Abstract]))) AND (("Nucleosides"[Mesh]) OR (((((((((((((((Nucleoside[Title/Abstract]) OR (Nucleotide analog*[Title/Abstract])) OR (Nucleoside analog*[Title/Abstract])) OR (NAs[Title/Abstract])) OR (NUC[Title/Abstract])) OR (Lamivudine[Title/Abstract])) OR (LAM[Title/Abstract])) OR (Adefovir[Title/Abstract])) OR (ADV[Title/Abstract])) OR (Entecavir[Title/Abstract])) OR (ETV[Title/Abstract])) OR (Telbivudine[Title/Abstract])) OR (TBV[Title/Abstract])) OR (Tenofovir[Title/Abstract])) OR (TDF[Title/Abstract])))) AND (("Interferons"[Mesh]) OR (((((Peginterferon[Title/Abstract]) OR (pegylated interferon[Title/Abstract])) OR (PEG-IFN[Title/Abstract])) OR (pegasys[Title/Abstract])) OR (peg interferon[Title/Abstract])))) AND ((((randomized controlled trial[Title/Abstract]) OR (randomized[Title/Abstract])) OR (random*[Title/Abstract])) OR (placebo[Title/Abstract])) Sort by: Most Recent | 241 |

Table 2 Embase

| No. | Content | Result |
| --- | --- | --- |
| #1 | 'hepatitis b, chronic'/exp OR 'hepatitis b, chronic' OR 'hepatitis b'/exp OR 'hepatitis b virus'/exp OR 'chronic hepatitis b virus infection':ab,ti OR 'chronic hepatitis b':ab,ti OR 'hepatitis b virus infection, chronic':ab,ti OR 'hepatitis b virus infection':ab,ti OR 'chb':ab,ti OR 'hbv':ab,ti | 182,800 |
| #2 | 'nucleosides'/exp OR 'nucleosides' OR 'nucleoside':ab,ti OR 'nucleotide analog*':ab,ti OR 'nucleoside analog*':ab,ti OR 'nas':ab,ti OR 'nuc':ab,ti OR 'lamivudine':ab,ti OR 'lam':ab,ti OR 'adefovir':ab,ti OR 'adv':ab,ti OR 'entecavir':ab,ti OR 'etv':ab,ti OR 'telbivudine':ab,ti OR 'tbv':ab,ti OR tenofovir:ab,ti OR 'tdf':ab,ti | 590,990 |
| #3 | 'interferons'/exp OR interferons OR peginterferon:ab,ti OR 'pegylated interferon':ab,ti OR 'peg ifn':ab,ti OR pegasys:ab,ti OR 'peg interferon':ab,ti | 749,396 |
| #4 | 'randomized controlled trial'/exp OR 'randomized controlled trial' OR 'randomized controlled trial':it OR randomized:ab,ti OR random*:ab,ti OR placebo:ab,ti | 2,473,448 |
| #5 | #1 AND #2 AND #3 AND #4 | 923 |

Table 3 Cochrane Library

| No. | Content | Result |
| --- | --- | --- |
| #1 | (hepatitis b, chronic):ti,ab,kw OR (hepatitis b):ti,ab,kw OR (hepatitis b virus):ti,ab,kw OR (chronic hepatitis b virus infection):ti,ab,kw OR (chronic hepatitis b):ti,ab,kw OR (hepatitis b virus infection, chronic):ti,ab,kw OR (hepatitis b virus infection):ti,ab,kw OR (hbv):ti,ab,kw OR (chb):ti,ab,kw | 13,287 |
| #2 | (nucleosides):ti,ab,kw OR (nucleoside):ti,ab,kw OR (nucleotide analog*):ti,ab,kw OR (nucleoside analog*):ti,ab,kw OR (nas):ti,ab,kw OR (nuc):ti,ab,kw OR (lamivudine):ti,ab,kw OR (lam):ti,ab,kw OR (adefovir):ti,ab,kw OR (adv):ti,ab,kw OR (entecavir):ti,ab,kw OR (etv):ti,ab,kw OR (telbivudine):ti,ab,kw OR (tbv):ti,ab,kw OR (tenofovir):ti,ab,kw OR (tdf):ti,ab,kw | 11,659 |
| #3 | (interferons):ti,ab,kw OR (peginterferon):ti,ab,kw OR (pegylated interferon):ti,ab,kw OR (peg-ifn):ti,ab,kw OR (peg interferon):ti,ab,kw OR (pegasys):ti,ab,kw | 5,616 |
| #4 | #1 AND #2 AND #3 | 584 |

Table 4 Web of science

| No. | Content | Result |
| --- | --- | --- |
| #1 | ((((((((TS=(Hepatitis B, Chronic)) OR TS=(Hepatitis B)) OR TS=(Hepatitis B virus)) OR TS=(Chronic Hepatitis B Virus Infection)) OR TS=(Chronic Hepatitis B)) OR TS=(Hepatitis B Virus Infection, Chronic)) OR TS=(Hepatitis B Virus Infection)) OR TS=(CHB)) OR TS=(HBV) | 99,580 |
| #2 | (((((((((((((((TS=(Nucleosides)) OR TS=(Nucleoside)) OR TS=(Nucleotide analog*)) OR TS=(Nucleoside analog*)) OR TS=(NAs)) OR TS=(NUC)) OR TS=(Lamivudine)) OR TS=(LAM)) OR TS=(Adefovir)) OR TS=(ADV)) OR TS=(Entecavir)) OR TS=(ETV)) OR TS=(Telbivudine)) OR TS=(TBV)) OR TS=(Tenofovir)) OR TS=(TDF) | 110,336 |
| #3 | (((((TS=(Interferons)) OR TS=(Peginterferon)) OR TS=(pegylated interferon)) OR TS=(PEG-IFN)) OR TS=(PEG-IFN)) OR TS=(peg interferon) | 26,387 |
| #4 | ALL=(randomized controlled trial) OR ALL=(randomized) OR ALL=(placebo) | 983,785 |
| #5 | #1 AND #2 AND #3 AND #4 | 255 |
